# Does a Common Pathway Transduce Symbiotic Signals in Plant–Microbe Interactions?

**DOI:** 10.3389/fpls.2016.00096

**Published:** 2016-02-16

**Authors:** Andrea Genre, Giulia Russo

**Affiliations:** Department of Life Sciences and Systems Biology, University of TurinTurin, Italy

**Keywords:** plant–microbe interactions, symbiosis, arbuscular mycorrhiza, legume nodulation, signaling pathways

## Abstract

Recent years have witnessed major advances in our knowledge of plant mutualistic symbioses such as the rhizobium-legume symbiosis (RLS) and arbuscular mycorrhizas (AM). Some of these findings caused the revision of longstanding hypotheses, but one of the most solid theories is that a conserved set of plant proteins rules the transduction of symbiotic signals from beneficial glomeromycetes and rhizobia in a so-called common symbiotic pathway (CSP). Nevertheless, the picture still misses several elements, and a few crucial points remain unclear. How does one common pathway discriminate between – at least – two symbionts? Can we exclude that microbes other than AM fungi and rhizobia also use this pathway to communicate with their host plants? We here discuss the possibility that our current view is biased by a long-lasting focus on legumes, whose ability to develop both AM and RLS is an exception among plants and a recent innovation in their evolution; investigations in non-legumes are starting to place legume symbiotic signaling in a broader perspective. Furthermore, recent studies suggest that CSP proteins act in a wider scenario of symbiotic and non-symbiotic signaling. Overall, evidence is accumulating in favor of distinct activities for CSP proteins in AM and RLS, depending on the molecular and cellular context where they act.

## Introduction

Our understanding of the major beneficial plant–microbe interactions – the rhizobium-legume symbiosis (RLS) and arbuscular mycorrhizas (AM) – has changed over the last decade in the light of breakthrough discoveries on the role of hormones, the exchange of symbiotic signals, or the lifetime of intraradical structures (Gutjahr and Parniske, 2013; Oldroyd, 2013; Schmitz and Harrison, 2014). AM fungi were once believed to open their way across the root apoplast thanks to cell wall degrading enzymes: genomic sequencing (Tisserant et al., 2013; Lin et al., 2014) suggests this is not the case and cellular evidence (Genre et al., 2005, 2008; Rich et al., 2014) has shown that host cell responses are critical for fungal colonization. Similarly, rhizobium entry in root hairs has been ascribed to the action of bacterial enzymes (Gage, 2004; Robledo et al., 2008); nevertheless, evidence is accumulating in favor of a plant-driven meltdown of the wall surrounding the ‘infection chamber,’ which then expands into the growing infection thread as one semi-solid compartment, where bacteria proliferate and slide (Fournier et al., 2008, 2015).

**FIGURE 1:**
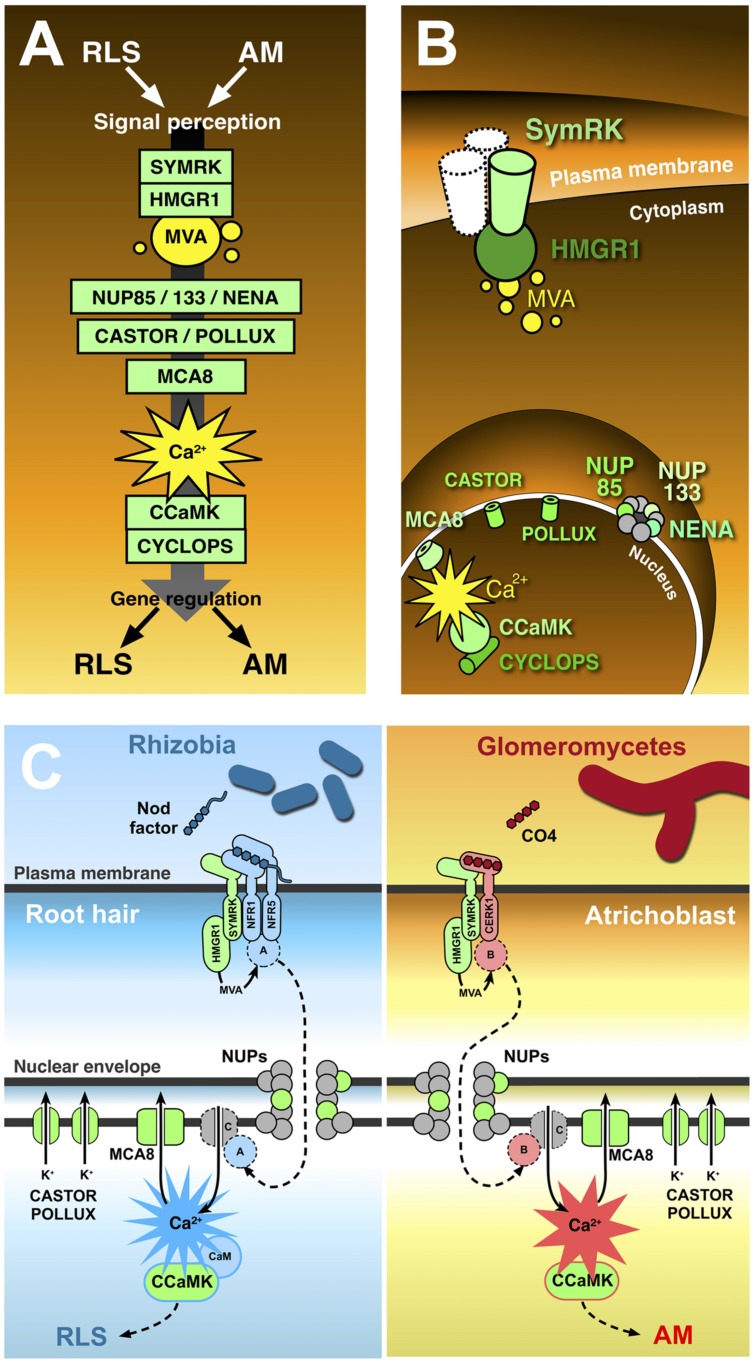
**The common symbiotic pathway.** Ten proteins (in green) have been identified as essential for both RLS and AM. In contrast with the conventional representation of signal transduction along the common symbiotic pathway **(A)**, CSP protein location in different plant cell districts **(B)** highlights the existence of significant discontinuities between three subsets of CSP members. The receptor-like kinase SYMRK is believed to interact with other co-receptors (such as NFR1 and NFR5) at the cell membrane; its cytoplasmic domain binds the mevalonate (MVA) biosynthetic enzyme HMGR1(3-Hydroxy-3-Methylglutaryl CoA Reductase 1), linking SYMRK activation to MVA synthesis in the vicinity of the plasmalemma. Three nucleoporins (NUP85, NUP133, and NENA) localize at the nuclear pore complex, but their role in signal transduction as well as their functional connection with the previous set of CSP proteins remains unclear. The final stretch of the CSP localizes to the nucleoplasm: here, repeated oscillations in Ca^2+^ concentration (spiking) are generated with the contribution of MCA8 (a nuclear envelope-bound ATP-powered Ca^2+^ pump) and the potassium channels encoded by *CASTOR* and *POLLUX* genes. Such Ca^2+^-mediated signals are believed to activate the nuclear kinase CCaMK. In turn, CYCLOPS phosphorylation by CCaMK preludes to the regulation of gene expression. **(C)** Presents a proposal model of receptor complex assortment and CSP protein role in two separate pathways for the transduction of Nod factors or chito-tetraose in legume root hairs and atrichoblasts, respectively. Localized on the plasma membrane of root hairs (left), the extracellular LysM domain of NFR1 and NFR5 directly bind Nod factors (Broghammer et al., 2012). NFR1 and NFR5 form a complex with SYMRK (Ried et al., 2014) and HMGR1 (Kevei et al., 2007). MVA, produced upon HMGR1 activation (Venkateshwaran et al., 2015) is small enough to diffuse through the nuclear pore complex without the involvement of nucleoporins (NUPs); nevertheless, the inclusion of three NUPs in the CSP opens the possibility that at least one additional unknown protein **(A)** is involved, which could be activated as a consequence of MVA production and translocated to the nucleus through the action of NUPs. Ca^2+^ spiking activation in the nucleoplasm is generated by the recursive release of Ca^2+^ through unidentified channel proteins **(C)** in the nuclear envelope, combined with the continuous action of ATP-powered Ca^2+^ pumps like MCA8 (Engstrom et al., 2002; Capoen et al., 2011). To consider a minimal number of unidentified proteins, we can assume Ca^2+^ channels are directly activated by nuclear-imported A. CASTOR/POLLUX has been proposed to act in concert with Ca^2+^ channels (Oldroyd, 2013). The resulting Ca^2+^ spiking activates CCaMK through a RLS-specific conformational change requiring calmodulin (Shimoda et al., 2012; Poovaiah et al., 2013), which then modulates the activity of gene expression regulators, allowing the establishment of RLS (Oldroyd, 2013). A parallel pathway acts in atrichoblasts (right), where chito-tetraose (CO4) released by glomeromycetes is recognised by a complex possibly including CERK1 (Miyata et al., 2014), SYMRK, and HMGR1. Also in this case an additional protein **(B)** is proposed to be activated by MVA. B is then translocated to the nucleoplasm, where it activates Ca^2+^ spiking signals with a distinct, AM-specific signature (Kosuta et al., 2008; Russo et al., 2013). Consequently, CCaMK is activated in an AM-specific mode (Shimoda et al., 2012; Poovaiah et al., 2013), and its activity regulates AM-specific gene expression.

The demonstration that host plants have major control over such interactions has supported the results of genetic studies, where single plant gene mutations were shown to block both bacterial and fungal penetration of the root (Kistner et al., 2005). Such studies on legume mutants gave rise to the hypothesis that AM and RLS share one signal transduction pathway (Oldroyd, 2013). This common symbiotic pathway, or CSP, is proposed to act downstream of both fungal and rhizobial signal perception and upstream of the activation of the appropriate response to either symbiont (**Figure 1**).

## The Common Symbiotic Pathway

Research on plant symbioses has largely been focussed on legumes. The culturability of rhizobia and their amenable genetics – compared to far less manageable glomeromycetes – is probably the main reason why research has progressed more rapidly in the field of RLS than AM. When our knowledge of RLS was later applied to AM, legumes were the obvious biological system for such studies.

A number of legume mutants had been selected for their RLS-defective phenotype (Catoira et al., 2000; Sandal et al., 2006); some of them were later found to display a mycorrhizal phenotype too, either blocking fungal entry at the epidermis surface or altering fungal development inside the root tissues (Kistner et al., 2005; Parniske, 2008; Oldroyd, 2013). The corresponding genes have been characterized and, based on their functions, positioned along a signal transduction pathway, the CSP, transducing glomeromycotan or rhizobial signal perception from the plasma membrane into the nucleus (**Figure 1**).

In *Lotus japonicus*, CSP gene products include the receptor-like kinase SYMRK; three nucleoporins, NUP85, NUP133, and NENA; CASTOR and POLLUX, cationic channels located on the nuclear envelope; a nuclear calcium- and calmodulin-dependent kinase CCaMK; and a CCaMK substrate, CYCLOPS (Oldroyd, 2013 and references therein). Furthermore, HMGR1, a key enzyme in the mevalonate biosynthetic pathway, and MCA8, a SERCA-type Ca^2+^-ATPase localized on the nuclear envelope, have been characterized in *Medicago truncatula* as additional members of the CSP (Kevei et al., 2007; Capoen et al., 2011). Secondary messengers such as mevalonate and Ca^2+^ have also been demonstrated to act within the CSP, either as a product of HMGR1 or an activator of CCaMK, respectively (Levy et al., 2004; Venkateshwaran et al., 2015).

In spite of its reassuring name, though, not all evidence confirms that CSP genes actually encode a signal transduction pathway that is common and restricted to RLS and AM.

### Pathway

While a solid link between signal perception and gene expression is a frequent feature in CSP representations (**Figure 1A**), evidence only supports a connection between sub-sets of the CSP members (**Figure 1B**).

A first set of CSP proteins includes the membrane-bound receptor-like kinase SYMRK (interacting with other proteins, like the Nod factor receptors NFR1 and NFR5) and the enzyme HMGR1 (Madsen et al., 2003; Kevei et al., 2007; Lefebvre et al., 2010). As a consequence of HMGR1 activation, mevalonate production can also be localized in the vicinity of the cytoplasmic face of the plasma membrane (Venkateshwaran et al., 2015).

A second cluster of CSP proteins is located in the nuclear pore complex: it is composed of three nucleoporins (NUP133, NUP85, and NENA), each of which is responsible for a strong symbiotic phenotype (Kanamori et al., 2006; Saito et al., 2007; Groth et al., 2010). This suggests that the nuclear pore must be controlling the import of an unknown key CSP component. This is not likely to be mevalonate, whose small size should allow nucleoporin-independent diffusion across the nuclear pore (Evans et al., 2004).

Also bound to the nuclear envelope are the ATP-powered Ca^2+^ pump MCA8 (Capoen et al., 2011) and the cationic channel encoded by CASTOR and POLLUX genes in *L. japonicus* (Ané et al., 2004; Charpentier et al., 2008). Both proteins contribute to the intense oscillations in nuclear Ca^2+^ concentration (spiking) that are observed during both AM and RLS establishment (Ehrhardt et al., 1996; Kosuta et al., 2008; Chabaud et al., 2011; Sieberer et al., 2012). In detail, so far unidentified channels are hypothesized to release Ca^2+^ from the nuclear envelope lumen. This release is sustained by the opposite flow of potassium ions (K^+^) through CASTOR/POLLUX, in a charge compensation mechanism (Parniske, 2008; Venkateshwaran et al., 2012); concomitant MCA8 activity contributes to the re-establishment of basic nuclear Ca^2+^ concentration at the end of each peak. Intriguingly, the nuclear pore has been proposed to play a role in flipping membrane-bound proteins from the outer to the inner nuclear membrane (Capoen et al., 2011), shedding light on the possible function of CSP nucleoporins in the targeting of CASTOR, POLLUX, MCA8, and Ca^2+^ channels.

The last group of CSP proteins whose direct interaction has been demonstrated resides in the nucleoplasm: Ca^2+^ spiking is supposed to activate CCaMK with the help of calmodulin, through a complex conformational change (Shimoda et al., 2012; Miller et al., 2013; Poovaiah et al., 2013); the enzyme can thus phosphorylate CYCLOPS, a CCaMK-interacting protein (Yano et al., 2008). Phosphorylated CYCLOPS regulates gene expression either directly, as in the case of the NIN promoter (Singh et al., 2014) or through the action of other transcription factors like NSP1, NSP2, and RAM1 (Oldroyd, 2013).

This topological review of the CSP highlights the gaps that uncouple each set of proteins from the next one: with so much missing information, depicting signal transduction along the CSP requires some extrapolation.

On the front of secondary messengers, reactive species of oxygen (Salzer et al., 1999; Pauly et al., 2006) and nitrogen (Meilhoc et al., 2010; Calcagno et al., 2012; Zhang et al., 2013) have also been associated with RLS and AM signaling, although their role in relation to the CSP remains unclear. Furthermore, growing evidence hints at the existence of symbiotic signal transduction pathways that bypass or run parallel to the CSP (Gutjahr et al., 2008, 2009; Bonfante and Requena, 2011), indicating that the plant’s perception of rhizobia and glomeromycetes could rely on multiple signaling routes.

In this context, the information we are still missing will critically challenge the CSP hypothesis: new data will either demonstrate that the CSP is indeed a pathway, or show that the remaining elements differ for each interaction, and what we had imagined as a straight line is rather a core of conserved, yet disconnected, protein functions.

### Symbiotic

Even if the concept of symbiosis can be extended to any interaction between organisms that live together (De Bary, 1879), the CSP concept is mostly restricted to RLS and AM, where the requirement for CSP genes was initially described (Stougaard, 2001; Kistner et al., 2005). Nevertheless, a third symbiosis also requires *SYMRK* (Gherbi et al., 2008), nuclear Ca^2+^ signals (Granqvist et al., 2015; Chabaud et al., 2016) and *CCaMK* (Svistoonoff et al., 2013): nitrogen-fixing actinorrhizas (**Supplementary Figure S1**).

In addition, non-symbiotic interactions have been shown to depend on CSP members. Parasitic interactions with root-knot nematodes involve *NFR1*, *NFR5*, and *SYMRK* (Weerasinghe et al., 2005). An intriguing role for *SYMRK* and *CCaMK* was also described during the colonization of *Pisum sativum* by the parasitic plant *Orobanche crenata* (Fernández-Aparicio et al., 2009): in this case *symrk* and *ccamk* mutants were more severely infected, indicating an unprecedented role for these CSP genes in inhibiting (rather than allowing) root colonization.

Furthermore, a key role in the regulation of gene expression has been demonstrated for *M. truncatula CCaMK* during interaction with the rhizobacterium *Pseudomonas fluorescens* (Sanchez et al., 2005).

The infection of *M. truncatula* by the pathogens *Phytophthora palmivora* (Huisman et al., 2015; Rey et al., 2015), *Aphanomyces euteiches*, and *Colletotrichum trifolii* (Rey et al., 2013) has been proposed to partially depend on Nod factor receptors, albeit the involvement of the CSP core was not highlighted. Nevertheless, *M. truncatula* mutants in *CCaMK* ortholog *DMI3* did not develop any cytoplasmic aggregation – a common defense response – upon *Phoma medicaginis* or *C. trifolii* attack (Genre et al., 2009), and displayed an anticipation of necrotrophic fungal growth.

Lastly, CSP homologs are found in mosses, Charophytes and Chlorophytes clades (Wang et al., 2010; Delaux et al., 2013b, 2015): the lack of known naturally occurring symbiotic interactions in such organisms (Field et al., 2015), and the tight phylogenetic relationship between Charales and land plants, might suggest the existence of conserved non-symbiotic functions for CSP proteins throughout the plant clade.

Overall, as research explores additional aspects of plant interactions, the functions of CSP genes appear to be growing in diversity, and extending well beyond the range of symbioses (**Supplementary Figure S1**).

### Common

There is no question CSP genes are essential for both legume endosymbiosis, but can we conclude that the signal transduction process involving CSP proteins is shared? Over the years, such a hypothesis has raised several questions (Bonfante and Requena, 2011), the most striking being: how can one pathway discern two signals, and activate distinct sets of downstream responses?

The foundations of the CSP hypothesis are built on the categorical results of forward genetic approaches: mutant phenotyping has shown that each CSP protein is indispensable for both RLS and AM establishment (Kistner et al., 2005). Nevertheless, signal transduction is not just a matter of protein presence/absence; finer aspects, such as the intensity of enzyme activation in response to each symbiont, could not be revealed by genetic investigations. In fact, recent biochemical analyses indicate that calmodulin binding is dispensable for CCaMK activation during mycorrhization, but essential for nodulation, suggesting that CCaMK is activated in two distinct modes during the perception of rhizobial versus glomeromycotan signals (Shimoda et al., 2012; Poovaiah et al., 2013). Along the same lines, evidence is accumulating in favor of different ‘signatures’ in the Ca^2+^-mediated signals, which can be responsible for such differential activation of CCaMK at the core of the CSP (Kosuta et al., 2008; Russo et al., 2013).

These studies strongly suggest that the same molecular actors can be playing different biochemical scripts in each interaction. The case of the GRAS-type transcription factor NSP2 (Kaló et al., 2005) is enlightening: NSP2 has been proposed to form a transcription regulator complex with other RLS- or AM-specific factors (NSP1 and RAM1, respectively); it has therefore been described as a CSP protein (Oldroyd, 2013). Nevertheless, *in vitro* interaction experiments support a model where NSP2 performs distinct symbiotic functions, depending on its interactors (Maillet et al., 2011; Gobbato et al., 2012, 2013; Lauressergues et al., 2012).

Importantly, cellular investigations on model legumes have demonstrated that rhizobia preferentially attach to and penetrate through root hair cells (Gage, 2004), whereas AM fungi contact and enter non-hair cells (atrichoblasts; Genre et al., 2005). Consequently, the study of early plant responses to rhizobial and glomeromycotan signaling only has a biological meaning when this specialization in root epidermal cell types is taken into account: while one pathway including all the necessary proteins could theoretically be designed based on legume genomes, it is likely that each cell type complements the expression of CSP genes with a set AM- or RLS-specific proteins, assembling two spatially and functionally distinct pathways (**Figure 1C**).

## Providing Context to CSP Proteins

Even if legumes remain the most important model plants for the study of symbiotic interactions, a growing number of publications is providing significant advancements on AM signal perception in non-legumes (Gutjahr et al., 2009; Miyata et al., 2014; Sun et al., 2015; Zhang et al., 2015).

A particular interest has recently been raised by rice mutants in *CERK1* (Miya et al., 2007; Sánchez-Vallet et al., 2015), a well-characterized LysM-type chitin receptor involved in defense responses: *cerk1* mutants display a strong mycorrhizal phenotype, blocking AM hyphae at the surface of the root epidermis (Miyata et al., 2014). RNAi-based knock-down of *CERK1* also induced a significant reduction in AM colonization (Zhang et al., 2015). Involving chitin receptors in symbiotic signaling is very intriguing: chitin-based molecules secreted by AM fungi activate the CSP and downstream responses (Maillet et al., 2011; Czaja et al., 2012; Genre et al., 2013; Giovannetti et al., 2015; Sun et al., 2015). Such Myc factors include lipo-chito-oligosaccharides (LCOs), structurally similar to Nod factors (Maillet et al., 2011), and short undecorated chito-oligosaccharides, or COs (Genre et al., 2013). LCOs appear to be particularly active in legumes, where they trigger a range of responses that are generally common to Nod Factor perception, such as lateral root formation or gene regulation (Maillet et al., 2011; Czaja et al., 2012; Sun et al., 2015), suggesting that some degree of overlap exists between legume perception of Nod and Myc factors. By contrast COs trigger CSP-dependent Ca^2+^ spiking in both legumes and non-legumes at concentrations as low as 10^-8^ M (Genre et al., 2013; Sun et al., 2015) and can be considered universal pre-symbiotic AM signals (Sun et al., 2015; Zhang et al., 2015).

Overall, a model is emerging where the assembly of different membrane-residing receptor complexes (Oldroyd, 2013; Liang et al., 2014; Gobbato, 2015; Limpens et al., 2015; Shinya et al., 2015) depends on which receptors are expressed by each cell type and possibly which signaling molecule is present. In the case of defense responses to pathogenic fungi, CERK1/CERK1 (as in *Arabidopsis*) or CERK1/CEBIP receptor dimers (as in rice) are hypothesized to bind long oligomers such as chito-octaose (CO8), half of the CO8 molecule fitting in to each receptor’s LysM domain (Wan et al., 2008; Shimizu et al., 2010; Liu et al., 2012; Shinya et al., 2015). The perception of shorter COs such as chito-tetraose could rather rely on monomeric receptors (Miyata et al., 2014; Shinya et al., 2015). In legumes, a further level of specificity results in their ability to respond to COs as well as LCOs (such as Nod factors), which fits with the proliferation of legume LysM and LysM-related receptor families through gene duplication events (Zhang et al., 2007).

It is reasonable to speculate that each receptor complex interacts with a corresponding set of cytoplasmic proteins (Asai et al., 2002). At present, information is very limited, but in analogy to SYMRK interaction with HMGR1, we can hypothesize that other proteins associate with each receptor, generating a signal-specific composition in the cytoplasmic moiety of the signaling complex. MAP kinases (Chen et al., 2012) may be playing a role; sensitivity to mevalonate appears as a stringent requisite for the selection of other candidates.

In such a scenario, CSP proteins would represent a conserved backbone in distinct AM and RLS signaling pathways that legumes localize in different cell types: atrichoblasts or root hairs, respectively. Symbiont-specific signal transduction and downstream responses would rather depend on the specific subsets of proteins that act in association with CSP members in each cell, as hypothesized in the schemes of **Figure 1C**.

## Conclusion

The history of life is rich in examples of so-called evolutionary tinkering: processes in evolution where new functions are obtained through small modifications of a pre-existing biological mechanism. Evolutionary tinkering consists of two opposite processes: on the one hand, gene duplication and neo-functionalization produce new proteins for the novel functions; on the other hand, all those genes that play the same function in both conditions are conserved. The hypothesis that RLS has evolved by redirecting AM responses toward bacterial accommodation is widely accepted and explains the numerous similarities that exist between these two interactions (Bonfante and Genre, 2008; Parniske, 2008). In particular, several features of plant cell restructuring (e.g., symbiotic interface biogenesis) are strikingly similar (Parniske, 2008). In this context, conserved genes should be much more numerous than just the few currently listed in the CSP; not surprisingly, *common symbiotic* genes have already been identified which do not fit into the *pathway*. To mention just a few examples, VAPYRIN (Pumplin et al., 2010) is a partially characterized protein featuring a Major Sperm Protein domain and several ankyrin repeats, likely involved in membrane dynamics; CERBERUS (Yano et al., 2009) is an E3 ubiquitin ligase. Both are required for symbiont accommodation, but more likely in cellular remodeling and interface development than in signaling. On the same line, a group of SNARE proteins belonging to the VAMP72 family has been involved in symbiotic interface assembly for both interactions (Ivanov et al., 2012). It is reasonable to conclude that CSP proteins belong to this array of conserved genes, and act in a complex mix of common and interaction-specific processes, required for the establishment of each symbiosis.

Further indications may come from detailed analyses of AM phenotypes in available mutants. The legume transcription factor *NSP1* was originally described as indispensable for rhizobial, but not fungal, colonization (Catoira et al., 2000; Smit et al., 2005). Nevertheless, a recent study showed that *nsp1* mutation significantly slows down AM infection (Delaux et al., 2013a). Similarly, a partial involvement of Nod factor receptors *NFR1* and *NFR5* has been described in the induction of common symbiotic responses such as root branching and gene regulation (Maillet et al., 2011; Czaja et al., 2012; Zhang et al., 2015). Such studies suggest that fine phenotypic analyses, reaching cellular and molecular detail, can be crucial for completing the picture.

In conclusion, the CSP lives on as a precious genetic reference in our simplistic models of plant-microbe signaling. Nevertheless, we have sufficient clues to suspect the existence of a more complex scenario of CSP protein localization and activity. Studying symbiotic signaling in non-legumes appears today as a very promising approach to address such questions (Watts-Williams and Cavagnaro, 2015): working on a biological system that intrinsically excludes the cross-talk of two evolutionarily related interactions such as RLS and AM will deliver crucial knowledge that can then be applied to decipher the multiple symbiotic system of legumes.

## Author Contributions

AG conceived the general layout of the manuscript and was primarily involved in text writing and figure preparation. GR contributed to text writing, and gave a major contribution to literature search and figure elaboration. Both AG and GR contributed to critical literature reviewing and model elaboration.

## Conflict of Interest Statement

The authors declare that the research was conducted in the absence of any commercial or financial relationships that could be construed as a potential conflict of interest.

## Supplementary material

FIGURE S1 **The role of CSP proteins in different plant interactions.** CSP and CSP-related proteins have been shown to play several roles beyond RLS and AM. SYMRK is required for the establishment of actinorhizal symbiosis and the parasitic interaction with root knot nematodes; furthermore it has been implicated in host defense against root colonization by parasitic plants. *CCaMK* is also necessary for actinorhizal symbiosis and defense responses to parasitic plants; in addition, it has a role in responses to pathogenic fungi. Beside CSP proteins, a few receptors that act upstream of the CSP have also been assigned with multiple roles. Nod factor receptors *NFR1* and *NFR5* are required for the establishment of actinorhizas and root knot nematode parasitism, and both have also been assigned a limited role in AM signaling. Finally, the chitin receptor *CERK1* is required for both defense responses to pathogenic fungi and the accommodation of AM fungi. Overall, a survey of the literature data suggests that CSP and CSP-related genes have a diverse spectrum of functions in several plant interactions.
