# Supplementary figures and images for: Does a Common Pathway Transduce Symbiotic Signals in Plant–Microbe Interactions?
Source: Front Plant Sci. 2016 Feb 16;7:96. doi: 10.3389/fpls.2016.00096 (PMC4754458; doi:10.3389/fpls.2016.00096)

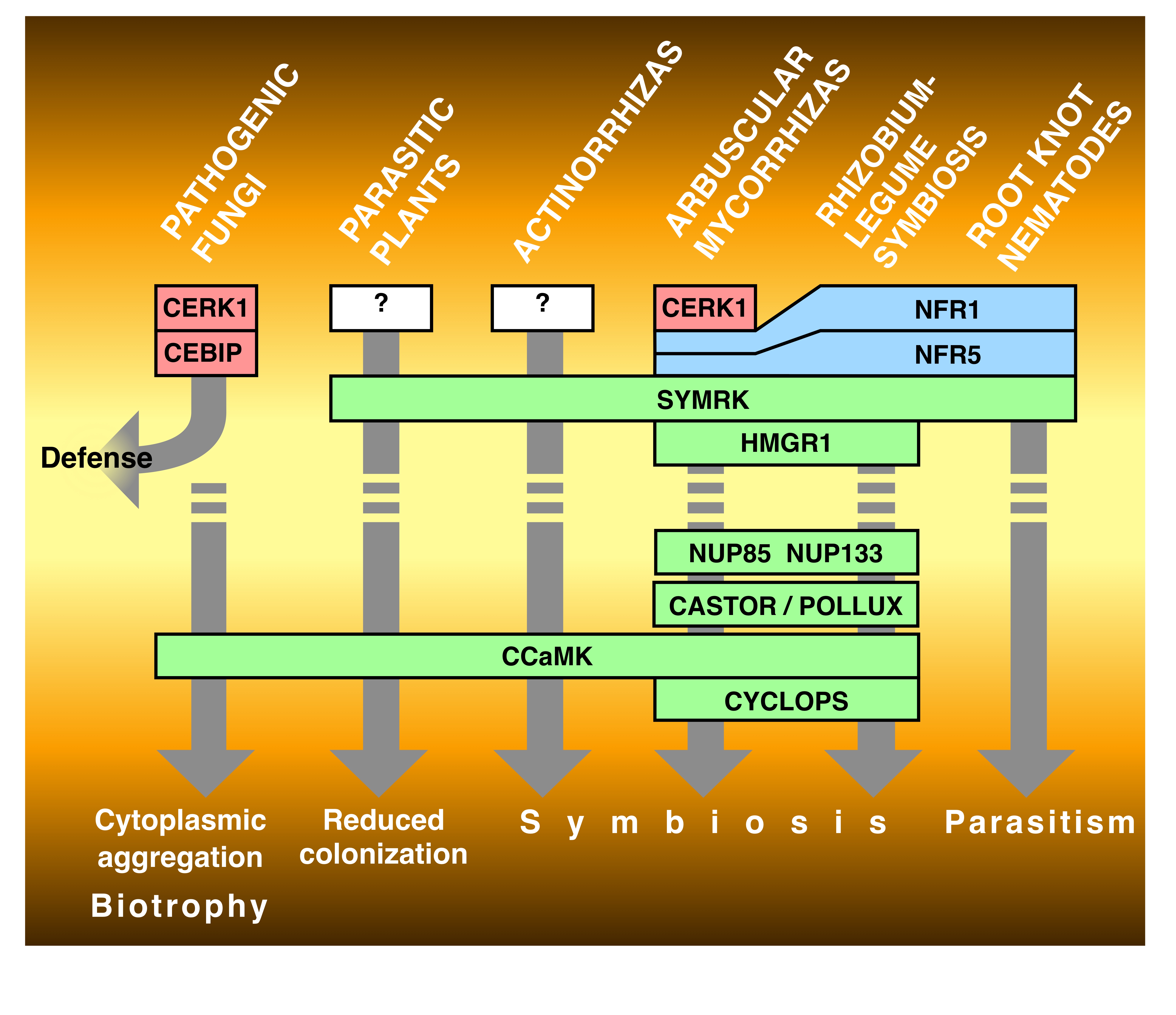

Supplement: FIGURE S1 — The role of CSP proteins in different plant interactions. CSP and CSP-related proteins have been shown to play several roles beyond RLS and AM. SYMRK is required for the establishment of actinorhizal symbiosis and the parasitic interaction with root knot nematodes; furthermore it has been implicated in host defense against root colonization by parasitic plants. CCaMK is also necessary for actinorhizal symbiosis and defense responses to parasitic plants; in addition, it has a role in responses to pathogenic fungi. Beside CSP proteins, a few receptors that act upstream of the CSP have also been assigned with multiple roles. Nod factor receptors NFR1 and NFR5 are required for the establishment of actinorhizas and root knot nematode parasitism, and both have also been assigned a limited role in AM signaling. Finally, the chitin receptor CERK1 is required for both defense responses to pathogenic fungi and the accommodation of AM fungi. Overall, a survey of the literature data suggests that CSP and CSP-related genes have a diverse spectrum of functions in several plant interactions. [file Image_1.JPG]
